# Supplementary material for: The genome sequence of the Northern Wall Brown, Lasiommata petropolitana (Fabricius, 1787) (Lepidoptera: Nymphalidae)
Source: Wellcome Open Res. 2025 Aug 28;10:462. [Version 1] doi: 10.12688/wellcomeopenres.24734.1 (PMC12749566; doi:10.12688/wellcomeopenres.24734.1)
Supplement: Peer review history

# Reviewer report: Reviewer response for version 1

**DOI:** 10.21956/wellcomeopenres.27251.r139455

**Published:** 2025-12-29

**Authors:** Paula Escuer Pifarré

**Affiliations:** 1 University of Neuchâtel, Neuchâtel, Spain

Here the authors present the genome assembly of a female individual of the Lepidoptera specie *Lasiommata petropolitana*. Haplotype 1 have a total size of 492 Mb aprox assembled in 30 chromosomal scaffolds including the W and Z sex chromosomes, as well as the mitochondrial genome, haplotype 2 is assembled at scaffold level. The paper is nicely written and methods are clear, following the standard assembly and annotation pipeline used for all the genomes from Psyche Project.

 Some minor comments:

 - in the introduction, at the sentence " *Lasiommata petropolitana* represents a distinct evolutionary significant unit (ESU) within the genus *Lasiommata*, showing low haplotype diversification relative to its congeners, which could be due to historical population bottlenecks, restricted gene flow, or a relatively recent divergence from its common ancestor ( Platania *et al.*, 2020) The species " there is a full stop missing before "The species".

 - I would recommend ending the introduction justifying what this genome could bring or be interesting for. (e. g. phylogenetics, genetic studies to develope conservation plans etc.)

Are sufficient details of methods and materials provided to allow replication by others?

Yes

Is the rationale for creating the dataset(s) clearly described?

Yes

Are the datasets clearly presented in a useable and accessible format?

Yes

Are the protocols appropriate and is the work technically sound?

Yes

Reviewer Expertise:

Genomics, Bioinformatics, Conservation

I confirm that I have read this submission and believe that I have an appropriate level of expertise to confirm that it is of an acceptable scientific standard.

# Reviewer report: Reviewer response for version 1

**DOI:** 10.21956/wellcomeopenres.27251.r139451

**Published:** 2025-12-23

**Authors:** Emily C Giles

**Affiliations:** 1 Cawthron Institute (Ringgold ID: 5732), Nelson, Nelson, New Zealand

The authors present a haplotype resolved genome assembly for the Northern Wall Brown, a butterfly. The rationale for generating this assembly is not well detailed beyond stating that it is evolutionarily different from other members of the genus. The non-butterfly researcher is left wondering why the genus as a whole is interesting and why resources should be used to generate assemblies for multiple species of this genus.

 A large proportion of the assembly (99%) falls into 30 pseudo chromosomes. The mitochondrial genome is also assembled. The authors used the same specimen for the PacBio HiFi library and the HiC library. They generated 1.2 million reads from the PacBio library and 752 million reads for the HiC library. The HiFi reads were assembled using Hifiasm in the Hi-C phasing mode. Scaffolding was done by mapping the HiC reads to the primary assembly and using YaHS to handle misassembles. The mitogenome was assembled using MitoHiFi. The assembly was decontaminated with the ASCC pipeline. This pipeline is nicely explained on the Sanger page that is linked, however it is noted that the pipeline is in a raw state of development. The pipeline developers indicate that the tool has been used on 500+ datasets, but one wonders what is the taxonomic breadth that this pipeline can handle? Prokaryotes, eukaryotes, microalgae to lions? Furthermore, is this pipeline applicable to genome assemblies of varying quality or only for PacBio HiFi primary assemblies? It would be nice to have this pipeline peer reviewed, but the description on the webpage is a great resource for the community. 

 The resulting genome assembly appears to be of high quality and contiguity. Heterozygosity does not appear to be overly low, despite the hypothesis that the species has experienced genetic bottlenecks. The coverage is not overly high (30X), but scaffold N50 is large and the contact map indicates that the assembly was well scaffolded. The sex chromosomes are large compared to the autosomes; this is interesting for researchers that don’t work with butterflies. Complete BUSCOs are extremely high and duplicated BUSCOs are very low. Overall, this looks like a great assembly.

Are sufficient details of methods and materials provided to allow replication by others?

Yes

Is the rationale for creating the dataset(s) clearly described?

Partly

Are the datasets clearly presented in a useable and accessible format?

Yes

Are the protocols appropriate and is the work technically sound?

Yes

Reviewer Expertise:

comparative genomics, population genomics, marine genomics, speciation

I confirm that I have read this submission and believe that I have an appropriate level of expertise to confirm that it is of an acceptable scientific standard.
